# A rare case of Lutembacher’s syndrome presenting with low-gradient severe mitral stenosis: case report and literature overview

**DOI:** 10.1093/ehjcr/ytae327

**Published:** 2024-07-10

**Authors:** Samah El-Mhadi, Belghait El Hajjaj, Nabil Laktib, Latifa Oukerraj, Mohamed Cherti

**Affiliations:** Department of Cardiology B, Ibn Sina University Hospital Center, Rabat, Morocco; Department of Cardiology B, Ibn Sina University Hospital Center, Rabat, Morocco; Department of Cardiology B, Ibn Sina University Hospital Center, Rabat, Morocco; Department of Cardiology B, Ibn Sina University Hospital Center, Rabat, Morocco; Department of Cardiology B, Ibn Sina University Hospital Center, Rabat, Morocco

**Keywords:** Lutembacher’s syndrome, Mitral stenosis, Atrial septal defect, Heart failure, Case report

## Abstract

**Background:**

Lutembacher’s syndrome refers to the rare combination of atrial septal defect and mitral stenosis. This condition is still underdiagnosed despite its distinct clinical, paraclinical, and therapeutic implications.

**Case summary:**

We report the case of a 60-year-old woman presenting with acute congestive heart failure. Investigations revealed the combination of low-gradient severe rheumatic mitral stenosis and ostium secundum atrial septal defect. Despite the medical team’s conservative management of her condition and recommendation for surgical intervention, the patient chose to discharge herself against medical advice. She died 6 months later.

**Conclusion:**

Lutembacher’s syndrome continues to be a rare condition in our practice. Once low-gradient severe mitral stenosis is diagnosed, it is essential to search for an associated atrial septal defect, especially when there is an early onset of significant right ventricular enlargement or dysfunction or when an intriguing inconsistency exists between the mitral area and the transmitral gradient.

**Learning points**

- Our case report underscores the importance of considering the possibility of coexisting conditions in patients with mitral stenosis. The discrepancy between the tight mitral valve area and the relatively low transmitral gradient raised suspicion for an additional pathology. The decision to perform a transoesophageal echocardiogram to characterize the presence of an atrial septal defect was pivotal.
- Our case report also highlights the need for a systematic approach and a high index of suspicion when interpreting diagnostic data in cases of valvular heart disease, especially when incongruities are observed between different parameters.

## Introduction

Lutembacher’s syndrome refers to the rare combination of atrial septal defect (ASD) and mitral stenosis (MS). This condition can be congenital or acquired, with the ASD typically being congenital and the MS often resulting from rheumatic heart disease. Although rare, it is essential to recognize this syndrome due to its distinct clinical, paraclinical, and therapeutic implications.^1^

## Summary figure

**Table:** 

Day 1	The patient was admitted to our cardiology department for management of acute congestive heart failure. Electrocardiogram showed atrial fibrillation and complete right bundle branch block. Chest X-ray revealed cardiomegaly with signs of atrial enlargement and pulmonary congestion. Transthoracic echocardiogram showed low-gradient severely compromised rheumatic MS. Laboratory results: significantly elevated brain natriuretic peptid. Management of the congestive heart failure and anticoagulation for atrial fibrillation.
Day 2	Transoesophageal echocardiogram confirmed the severe rheumatic MS and revealed an ostium secundum atrial septal defect with a left-to-right shunt.
Day 3	Mitral valve replacement, ASD patch closure, and tricuspid annuloplasty were recommended, but the patient declined. The patients was treated conservatively and discharged home.
After discharge	The patient died 6 months later.

## Case presentation

We report the case of a 60-year-old female patient admitted to the cardiology department for the management of acute congestive heart failure.

Upon admission, she presented with orthopnoea. Her heart rate was 100 b.p.m., blood pressure 90/50 mmHg, and oxygen saturation 89% on room air. Examination revealed bibasilar crackles in the lungs, oedema in the lower extremities extending to the loins, jugular venous distension, and ascites. Cardiovascular auscultation revealed a soft diastolic rumbling murmur in the mitral area suggestive of MS and a loud P2 in the pulmonary area.

**Figure 1:**
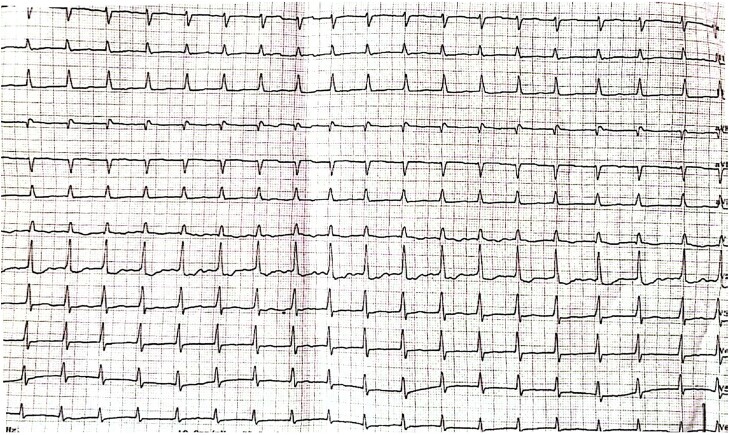
Electrocardiogram showing atrial fibrillation with ventricular rate at 100 b.p.m. and complete right bundle branch block.

The electrocardiogram showed atrial fibrillation with a ventricular rate of 100 b.p.m., right axis deviation, and complete right bundle branch block with secondary repolarization disorders (*Figure 1*).

**Figure 2:**
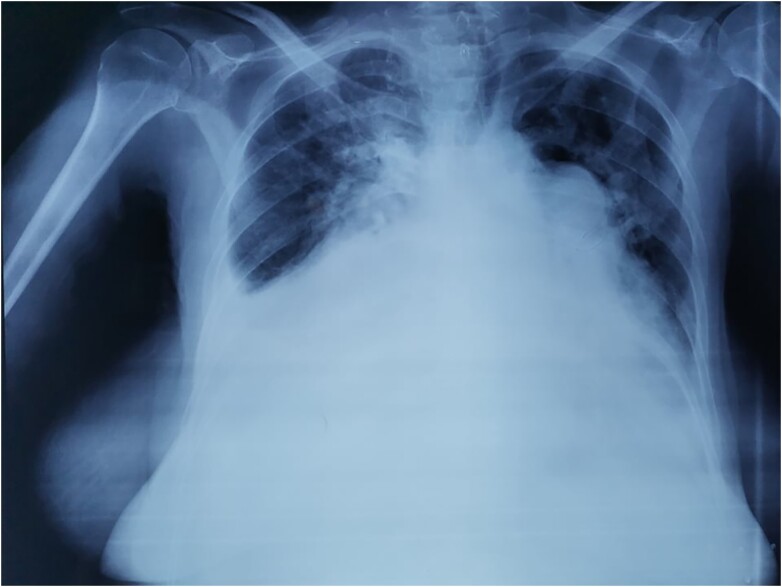
Chest X-ray showing cardiomegaly and pulmonary congestion.

Chest X-ray showed cardiomegaly with a straightening of the left heart border, a prominent pulmonary artery, right atrial enlargement with bilateral alveolar and interstitial infiltrates, and bilateral pleural effusion suggestive of pulmonary congestion (*Figure 2*).

**Figure 3:**
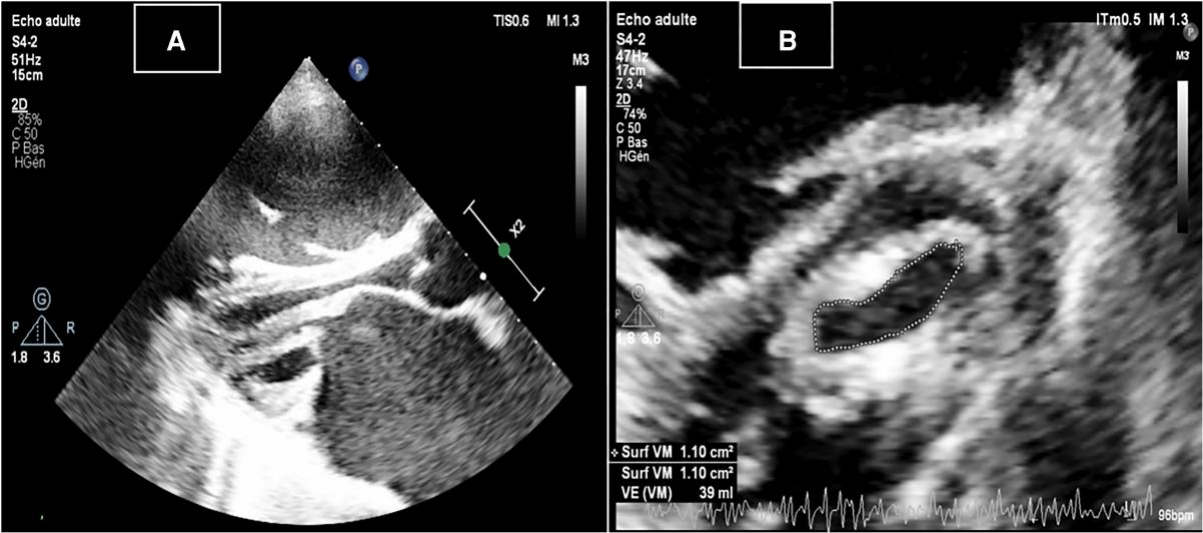
(*A*) Transthoracic echocardiogram parasternal long-axis view revealing thickened mitral leaflets with restricted opening in diastole. (*B*) Transthoracic echocardiogram parasternal short-axis of the mitral valve showing severe mitral stenosis with a mitral area of 1.1 cm^2^.

**Figure 4:**
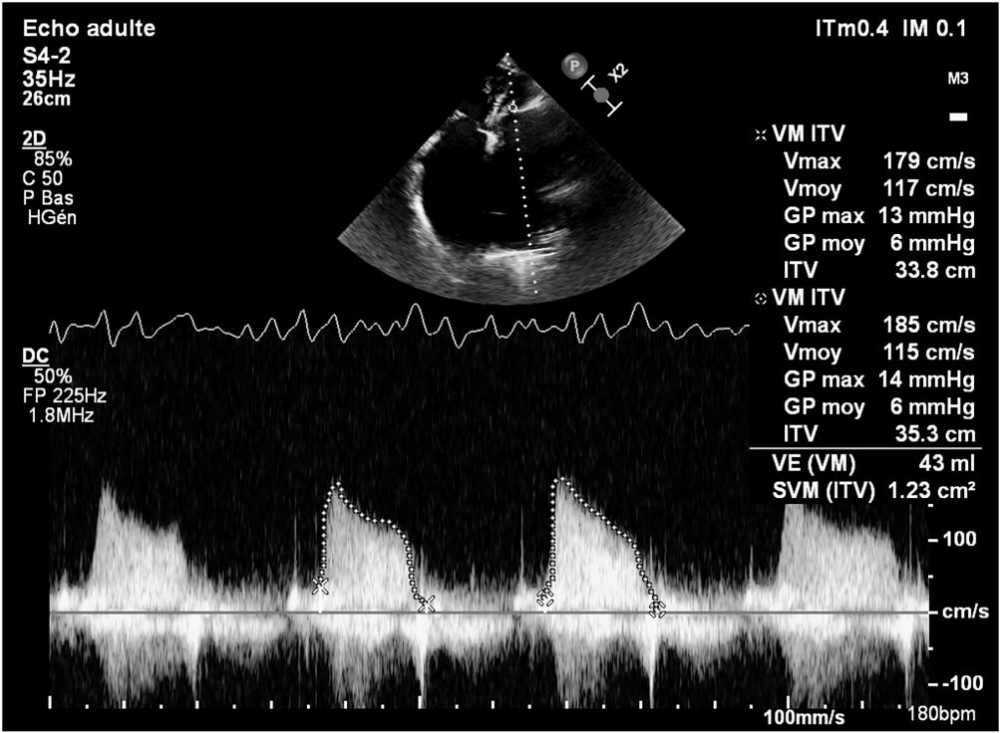
Transthoracic echocardiogram apical four-chamber view assessing the medium transmitral gradient, which measures 6 mmHg.

Transthoracic echocardiogram revealed thickened mitral valve cusps with commissural fusion and reduced opening, resulting in a mitral valve area of 1.1 cm^2^, indicative of severely compromised rheumatic MS, with a Wilkins score of 11 (*Figure 3*). The mean transmitral gradient was only 6 mmHg (*Figure 4*). The mitral regurgitation was classified as Grade 2 before the patient’s depletion with an effective regurgitant orifice (ERO) of 0.20 cm^2^ and a regurgitant volume (RV) of 30 mL and downgraded to Grade 1 after depletion. Atrial dilation wad noted. The right ventricle was also dilated with normal systolic function. The tricuspid regurgitation was moderate, with a vena contracta of 5 mm, a proximal isovelocity surface area radius of 7 mm, an ERO of 0.24 cm^2^, and a RV of 37 mL. The probability of pulmonary hypertension was high, with the pulmonary artery systolic pressure estimated at 75 mmHg through the tricuspid regurgitation flow (TR max velocity: 3.9 m/s and right atrial pressure estimated at 15 mmHg due to a dilated and non-compliant inferior vena cava). Left ventricular ejection fraction was preserved.

**Figure 5:**
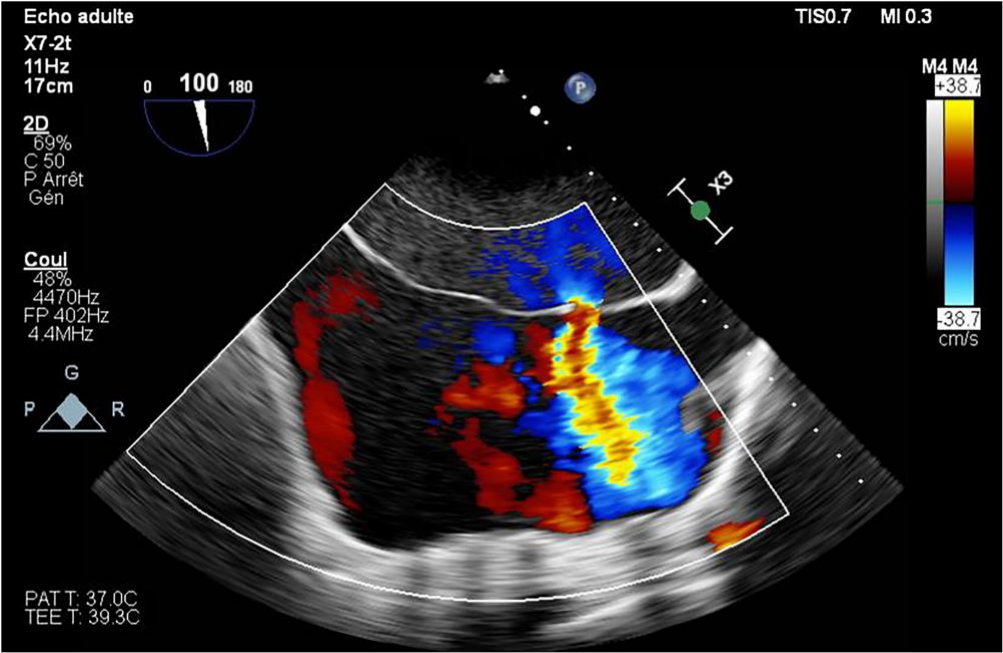
Transoesophageal echocardiogram bicaval view showing the shunt flow through the ostium secundum atrial septal defect.

Given the mismatch within the tight mitral area and the transmitral gradient, we decided to perform a transoesophageal echocardiogram. It confirmed the presence of severe rheumatic MS and revealed an associated ostium secundum type of ASD measuring 6 mm, with a left-to-right shunt (*Figure 5*).

Laboratory examination showed a significant elevation of brain-type natriuretic peptide (2036 pg/mL). The blood cell count, the routine liver and kidney function blood tests, and the C-reactive protein were all within the normal range.

The therapeutic approach for managing the congestive state involved initial oxygen therapy, the administration of furosemide at a dose of 120 mg/day intravenously with concurrent potassium replacement, and the introduction of spironolactone starting at 25 mg and then increased to 50 mg/day. Curative anticoagulation was initiated with warfarin at a dose of 5 mg/day due to valvular atrial fibrillation.

Although suggestions were made for mitral valve surgical replacement coupled with tricuspid annuloplasty and patch closure of the ASD, the patient declined this option. A percutaneous treatment was considered as a potentially beneficial alternative; however, the necessary technical platform was not available for implementation.

Right heart catheterization could have been useful as a part of the pre-operative assessment. However, since the patient declined surgical treatment, we deemed right heart catheterization unnecessary in the absence of a therapeutic plan.

As a result, the patient received conservative treatment and was discharged home. Despite optimal medical management, the absence of surgical intervention contributed to the deterioration of her condition.

The patient died 6 months after discharge.

## Discussion

*Lutembacher* was credited with the first description of the association of ASD with MS in 1916, even though an earlier description was found in a letter written by anatomist *Johann Friedrich Meckel* to *Albrecht Von Haller* in 1750.^1^ The definition of Lutembacher’s syndrome has undergone several changes, but the current consensus stipulates that both the ASD and the MS can be either congenital or acquired.^2^

Atrial septal defect constitutes one of the most common congenital cardiac abnormalities. However, acquired ones exist and may be due to infectious endocarditis and ischaemic or traumatic septal rupture.^3^

The natural history of ASD depends, among other considerations, on the coexistence of another heart disease such as MS.

Mitral stenosis is usually labelled as rheumatic, especially in developing countries such as Morocco, but can also be degenerative or secondary to infective endocarditis, granulomatous infiltration, nodular rheumatoid arthritis, or systemic lupus erythematosus.^4^

The combination of ASD and MS, known as Lutembacher’s syndrome, is probably fortuitous. In patients with ASD, the incidence rate of MS has been estimated at 4%, while the estimated incidence rate of ASD in patients with MS is 0.6–0.7%.^5^ Lutembacher’s syndrome shows a predisposition for women since MS and ASD are more frequently observed in women.^5^

In typical Lutembacher’s syndrome, the MS is acquired and rheumatic, and the ASD is congenital and of the ostium secundum type.^6^

The medical approach to patients with Lutembacher’s syndrome is quite complex. Clinical presentations and haemodynamic features vary depending on the severity of MS, the characteristics of ASD (anatomical type and size), the pulmonary vascular resistance, and the right ventricle compliance.^2^

When ASD is restrictive and MS stenosis is severe, the symptoms and clinical course resemble isolated MS of equivalent severity. However, interactions between non-restrictive ASD and MS are more complex.

Atrial septal defect has a series of clinical and haemodynamic effects when associated with MS.^2^ Atrial septal defect increases left-to-right shunt and reduces left ventricle filling and cardiac output, making fatigue more frequent. It also decompresses the left atrium and ameliorates symptoms of pulmonary congestion. It promotes right atrial enlargement and increases the incidence of atrial fibrillation but decreases the risk of left atrial thrombosis. Moreover, ASD may lead to early pulmonary artery hypertension and increases the incidence of right ventricle failure. It also increases the risk of paradoxical embolism.^2^

Management of patients with Lutembacher’s syndrome includes symptomatic treatment based on the administration of diuretics for pulmonary congestion and right heart failure, administration of beta-blockers for heart rate control, and curative-dose anticoagulation if atrial fibrillation and cardiac thrombosis are associated.

Open heart surgery has long been considered the standard therapy for Lutembacher’s syndrome, but thanks to the considerable developments in medical technology, transcatheter therapy combining balloon mitral valvuloplasty and ASD closure has been presented as an attractive alternative therapy.^7^ Most combined procedures are performed within the same setting, and mitral valvuloplasty is performed first while the ASD is used as a natural passage for mitral procedures. In cases with a high probability of mitral restenosis, cardiac surgery with valve replacement constitutes the best radical therapeutic option.^8^

## Conclusion

Lutembacher’s syndrome remains a relatively uncommon condition, often underdiagnosed in medical practice. When MS is diagnosed, the presence of an ASD should be actively sought, particularly in cases where significant right ventricular dilation and/or dysfunction occur early or when there is an intriguing mismatch between the mitral area and the transmitral gradient.

## Data Availability

No new data were generated or analysed in support of this article.

## Lead author biography

Samah El-Mhadi, M.D., is a cardiologist at Ibn Sina University Hospital Center in Rabat, Morocco.
